# Reviewing the Current State of Renal Cell Carcinoma with a Tumor Thrombus: Epidemiology, Pathophysiology, Metastasis, and Systemic Therapy

**DOI:** 10.1007/s11934-025-01301-4

**Published:** 2025-11-01

**Authors:** Maxwell Sandberg, Randall Bissette, Kimberly Waggener, Gaetano Ciancio, Alejandro R. Rodriguez

**Affiliations:** 1 Department of Urology, Wake Forest Baptist Medical Center, Winston Salem, NC 27101 USA; 2 Wake Forest University School of Medicine, Winston Salem, NC 27157 USA; 3 University of Miami Miller School of Medicine, Miami, FL USA

**Keywords:** Renal cell carcinoma, Tumor thrombus, Systemic therapy, Metastasis, Pathophysiology

## Abstract

**Purpose of Review:**

The purpose of this review is to highlight relevant literature on the epidemiology and pathophysiology of renal cell carcinoma with a tumor thrombus (RCC-TT), metastatic management, and the role of systemic therapy (ST).

**Recent Findings:**

Current research indicates different prevalences and presentations by geography, with many classification systems for RCC-TT. Pathophysiology remains poorly understood for TT formation. Some patients may benefit from cytoreductive nephrectomy with tumor thrombectomy (CN-TT) as well as ST administration, but this population is poorly defined.

**Summary:**

RCC-TT is a complex disease to manage and lacks high-powered publications relative to RCC without a TT. Future research should focus on the genetics behind TT formation and propagation, CN-TT outcomes, and which patients should receive ST and the optimal timing/dosage.

## Introduction

Kidney cancer is expected to represent approximately 4% of all cancer cases in the year 2025 [1]. Furthermore, it is estimated that approximately 81,000 new kidney cancer cases will be diagnosed in 2025 and 2% of all cancer-related deaths will be from the disease [1]. Kidney cancer is sexually dimorphic, afflicting men at greater rates than women [2]. There is also geographical bias in kidney cancer. The greatest incidence is in Europe, followed by Asia, and then North America (NA). The lowest incidences are found in Latin America, Africa, and Oceania [2]. Kidney cancer is a relevant genitourinary cancer to study for many reasons, but perhaps most importantly is its high mortality, estimated to have a five-year survival rate of just 79% [1].

While kidney cancer has multiple variants, most cases are renal cell carcinoma (RCC). RCC represents 90% of all kidney cancer [3]. The three most common subtypes of RCC are clear cell, papillary, and chromophobe [2, 4]. Anywhere from 3–10% of patients will be found to have RCC and a tumor thrombus (RCC-TT) at the time of diagnosis [5, 6]. A TT is any extension of tumor into a vessel [7]. For RCC, TT can extend from the renal vein, into the inferior vena cava (IVC), and all the way up to the right atrium of the heart. RCC-TT is relevant as it changes the staging of RCC and can significantly impact management [5, 7]. Treatment is necessary in virtually all instances, and the gold standard is surgical removal in the form of radical nephrectomy with tumor thrombectomy (RN-TT). Over the years, operative techniques have been perfected and are well published and described [5, 8–11]. Despite comprehensive publications on RCC-TT in the operative domain and patient outcomes, there is still a paucity of research in other areas of the disease necessitating discovery. Over the last three years, our international research group, the Intercontinental Collaboration on Renal Cell Carcinoma (ICORCC), has attempted to study RCC-TT, forming a large patient database spanning four continents [NA, Central/South America (CSA), Spain, and South Korea], and over 500 patients. Through this collaborative effort, we have identified specific areas of RCC-TT research that lack strong and comprehensive data. These include, but are not limited to RCC-TT epidemiology, pathophysiology, outcomes/prognosis, metastatic RCC-TT (mRCC-TT), and the role of systemic therapy (ST) in RCC-TT, all of which require further attention. The purpose of this narrative review is to summarize the literature on RCC-TT, focusing on epidemiology, pathophysiology, outcomes/prognosis, metastasis, and the role of ST.

## Epidemiology

**Fig. 1:**
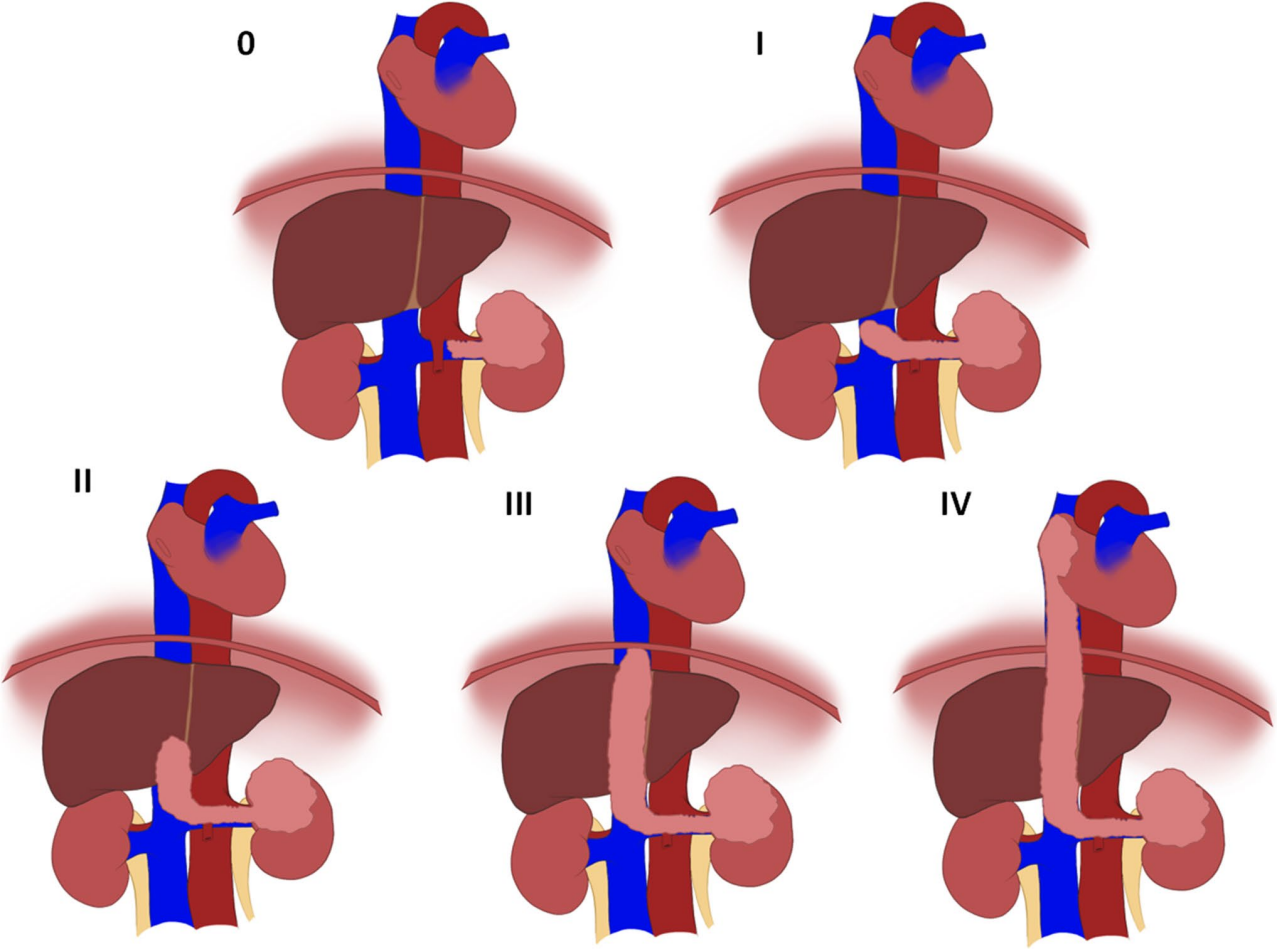
Tumor thrombus representations. The following images represent RCC-TT at levels 0-IV using the Neves and Zincke classification system. TT is included in each image going up to the corresponding anatomic level. Additional associated anatomy is also included

RCC-TT is classified by the level of TT. A landmark paper out of the Mayo Clinic by Neves and Zincke is still used by many as the gold standard classification system [12]. They consider four different levels of TT, with an additional level 0 TT limited to the renal vein only. Level I is a TT into the IVC but no more than 2 cm (cm) above the renal vein, Level II is a TT into the IVC and more than 2 cm above the renal vein, but not to the level of the hepatic vein, level III is a TT into the IVC and above the hepatic vein, but not to the level of the diaphragm, and lastly, level IV is a TT into the supradiaphragmatic IVC and/or right atrium [12]. Over the years, many other research groups have built off the original Mayo Clinic definition to form classification systems of their own. Table 1 displays the different major classification systems for RCC-TT and the specific qualifications required at each TT level, and Fig. 1 shows an anatomic diagram using the Mayo Clinic classification system of RCC-TT.

**Table 1:** Commonly used classification systems for tumor thrombus level

*Author (year)*	Neves and Zincke (1987) [12]	Libertino (2004) [51]	Mandhani (2015) [82]	Ciancio (2002) [83]
*Basis*	Anatomic: Relation to hepatic vein	Anatomic: Relation to diaphragm	Surgical: Need for clamping hepatoduodenal ligament	Level III subclassification, Anatomic: Relation to the hepatic vein
0	≤ Renal vein	–	–	
I	Into IVC but ≤ 2 cm above renal vein	Into IVC but < diaphragm	Caudal to insertion of hepatic vein	
II	> 2 cm above renal vein but < hepatic vein	Into IVC > diaphragm but < right atrium	Retrohepatic, suprahepatic infradiaphragmatic or at the suprahepatic supradiaphragamatic IVC	
III	Above hepatic vein but < diaphragm	Into right atrium	Into right atrium	IIIa: Infrahepatic veins reaching the retrohepatic IVC
				IIIb: To the hepatic veins
				IIIc: Suprahepatic veins but infradiaphragmatic
				IIId: Supradiaphragmatic but below right atrium
IV	Above diaphragm	–	–	

This table categorizes TT classification systems by publication author, with basis for classification system described

The original Neves and Zincke study was very limited in scope, at only 54 patients, but an additional paper out of the Mayo Clinic in 2004 included 540 patients using the same classification system. Level 0 made up about 65% of patients, level I made up around 12% of patients, level II about 14% of patients, level III nearly 5% of patients, and level IV was 4% of patients [13]. Martínez-Salamanca et al. conducted a multi-institutional consortium to identify the percentage of TT at each level in over 1,000 patients [6]. Interestingly, nearly 52% of their cohort had TT limited to the renal vein only, with 48% having TT in the IVC or higher. Kaptein et al. conducted a prospective study on 647 RCC patients, of which 86 were found to have RCC-TT [14]. They found nearly 40% of RCC-TT patients had a level 0 TT (extension only into renal vein), 43% had a level I-III TT (into the IVC but below the diaphragm), and approximately 17% of patients had TT extension beyond the diaphragm, labeled as level IV TT [14]. In a smaller study by Zisman et al. focusing on RCC-TT and the role of immunotherapy (IO), the authors identified 207 RCC-TT patients that underwent RN-TT, of which 48% had a TT limited to the renal vein and 52% had a TT beyond the renal vein [15]. Hatakeyama et al. performed a large retrospective analysis of 520 RCC patients in Japan from 1995–2014, of which 85 had RCC-TT graded according to the original Mayo Clinic classification system [16]. Most patients had a level 0 TT (49%), followed by level II (18%), level I (13%), level III (14%), and level IV (5%). In our own research group ICORCC, we have reported on 478 patients with RCC-TT [17]. The majority of patients (48%) had a TT ≤ level I using the Mayo Clinic classification system, followed by level II (22%), then level III (19%), and level IV (11%). Interestingly though, we identified geographical differences in TT level, with a greater TT level in patients receiving their care in CSA [17]. The exact prevalence at each classification level varies by the system to grade RCC-TT and study, but most evidence indicates around half of patients will have a TT limited to either the renal vein or IVC below the hepatic veins, with the smallest percentage of patients having TT extension above the diaphragm.

Another important epidemiologic question worth focusing on is the rate of mRCC-TT at diagnosis. The original Neves and Zincke paper noted a metastatic rate at one third of patients [12]. In a significantly larger cohort with over 1,000 patients, Martínez-Salamanca found 24% of patients had mRCC-TT [6]. An international consortium of 1,192 patients found the rate of mRCC-TT to be 27%, with similar rates of mRCC-TT based on whether TT was limited to the renal vein, IVC, or beyond [18]. In a smaller publication by Nesbitt et al. outlining surgical resection techniques, 32% (12/37) patients had mRCC-TT prior to RN-TT [19]. ICORCC’s research group identified greater rates of mRCC-TT at diagnosis in an international consortium, around 53% [17]. Kaptein et al. found TT at the time of RCC diagnosis predicted a threefold greater odds of having metastatic disease relative to RCC alone [14].

As with any epidemiologic analysis, geography must be accounted for. RCC-TT is significantly lacking in research of this domain. The limited research that does exist indicates significant inequities are present with respect to the distribution and type of tumors present in RCC-TT. Martínez-Salamanca and Wagner et al. both published data on large international consortiums studying RCC-TT [6, 18]. These groups pulled patients from Europe and NA. Most patients had a clear cell tumor subtype (~ 91–94%), followed by papillary (~ 2.5%), then chromophobe tumors (~ 2%), collecting duct tumors (~ 1.5%), and then other subtypes (~ 1–4%). Unfortunately, these groups lacked patient representation from other areas of the world, like Latin America and Asia. ICORCC published a comparison of RCC-TT between NA, CSA, and South Korea [17]. Similar percentages of clear cell tumors were found, with a slightly greater proportion of papillary tumors, around 6–8%, with no difference by geographic region of origin. Interestingly though, patients from CSA had a significantly greater proportion of sarcomatoid differentiation in this study relative to NA and South Korea [17]. Zhao et al. published a 144 patient cohort with RCC-TT from China, of which 22 (15%) patients had sarcomatoid differentiation [20]. Ultimately, this research highlights the importance of global health and health equity, which RCC-TT appears to be severely behind.

## Pathophysiology

### Genetic Risk

There are multiple theories behind the development of RCC, and evidence is much stronger compared to RCC-TT. Nabi et al. have published that around 95% of all clear cell RCC cases have a loss of the short arm of chromosome 3 [21]. The most frequently mutated gene in RCC is the VHL gene, a tumor suppressor [22]. This can be point mutations, insertions, deletions, chromosomal loss, or changes in methylation patterns. Acquired risks for RCC include smoking, obesity, hypertension, and renal disease [23]. Von Hippel Lindau disease is the most common genetic cause of RCC [24]. While a multitude of publications have focused on RCC pathophysiology, a paucity of literature has been devoted to RCC-TT.

A TT is the extension of tumor cells into the vasculature [25]. Evidence indicates that no mutations aside from those required for initial RCC development are needed for TT development. Interestingly though, the genomic profile of a primary RCC tumor and its TT often differ [25]. Kim et al. performed a prospective study involving histologic and genomic analysis of 83 RCC-TT patients scheduled for RN-TT [26]. The authors compared patients who did and did not develop mRCC-TT, finding different genomic profiles. Inflammation and mammalian target of rapamycin were upregulated in mRCC-TT. Monocyte counts and regulatory T cells were also greater in mRCC-TT patients [26]. TT have been also been found to display high rates of sarcomatoid and rhabdoid features, which are associated with poor CSS [25, 27]. It has also been reported that relative to matched primary tumors, RCC-TT has increased CD8 + T cells with a progenitor exhausted phenotype [28]. Further, protein expression differs in the TT relative to the primary tumor. Prostate specific membrane antigen (PSMA) is expressed at greater levels in TT relative to their primary RCC tumors in the kidney [27, 29]. This is relevant as PSMA is felt to be important in tumor neovascularization [27]. Immune cell dysfunction also likely plays a role in RCC-TT. Programmed cell death 1 receptor ligand (PD-L1) has been demonstrated to show differential expression between primary RCC’s and their associated TT, with lower expression in the TT [27, 30]. Given that PD-L1 is a common target of systemic therapy, this has clinical management implications. Preoperative absolute lymphocyte counts have also been shown to be greater in patients who die from RCC-TT compared to those who do not [31]. Additionally, lower lymphocyte to monocyte ratios have been implicated in mRCC-TT development [31].

### Acquired Risk

It is well-established that thrombosis secondary to cancer (cancer-associated thrombosis) is a frequent cause of morbidity and mortality in patients with cancer [32]. Aside from RCC, TT has been associated with diseases like hepatocellular carcinoma, pancreatic neuroendocrine tumors, and lung cancer [32]. Cancer cells cause inflammation, coagulopathies, and hypoxia to tissue [33]. These factors in concert lead to TT development. Cancer cells make tissue factor, which activates factor VII and then in turn factor X [33]. This is relevant as factor X is a central molecule in the coagulation cascade [34]. Cancer cells also secrete plasminogen activator inhibitor, which blocks fibrinolysis [33]. Further, inflammatory cytokines activate vascular endothelium, leading to additional platelet adherence and aggregation [33]. Blood is also static around the primary tumor, additionally predisposing patients to thrombus formation [32]. This combination leads to TT development. TT can lead to both free-floating tumor or actual invasion into the vascular wall [27]. This can often be seen on magnetic resonance imaging (MRI) preoperatively and vascular invasion can alter pathologic margins of vessels during RN-TT [27, 35].

### Other Associations

A TT is felt to disrupt normal vascular endothelium and blood flow, leading to venous stasis, which can lead to bland thrombus (BT) development, which is a different entity than a TT [32, 36]. BT appears uniquely different from TT on imaging. BT tends to be smooth and regular on imaging versus a TT that is irregular [37]. Further, enhancement patterns have been shown to differ between TT and BT [37]. The presence of a BT has been proven to complicate RN-TT, leading to greater operative times, more blood loss, and higher postoperative complications [37–39].

Many interesting research studies have identified important findings which have improved our understanding of TT development, and what makes RCC-TT different from RCC. The pathophysiology of RCC-TT is still not fully understood though, and much of the current literature has not been adequately translated from the basic science realm into clinical practice. In our opinion, future research should focus on ways to make this transition.

## Outcomes and Prognosis

The classic triad for RCC presentation is flank pain, hematuria, and flank fullness/palpable mass, yet only 10–15% of patients will present with all three [3]. Further, nearly 50% of RCC diagnoses are incidental as patients are asymptomatic [3]. Far less information exists on RCC-TT presentation. Best evidence still seems to indicate most patients are asymptomatic when diagnosed [40]. Nevertheless, this is not to say that the classic triad does not apply to RCC-TT, as a minority of patients are often still cited as having symptoms like hematuria or flank pain at diagnosis [41]. Skinner et al. noted 41% of their 56 patient cohort with RCC-TT undergoing RN-TT had symptoms of obstruction of the IVC like abdominal pain, lower back pain, leg swelling, and weight gain [42]. An absence of obstructive symptoms appears to be fairly common in RCC-TT, because of venous collateral growth off the IVC [5]. Constitutional symptoms like weight loss, night sweats, and fever are rare, noted around 5–15% [40, 43]. Specific TT symptoms often cited in the literature include right heart failure, lower extremity edema, dilated superficial abdominal veins, ascites, Budd-Chiari syndrome, pulmonary embolism, and varicocele [40, 44–48]. Interestingly, Skinner et al.’s study estimated an incidence of varicocele in RCC-TT patients of 30–40%, though additional confirmatory studies backing this claim are lacking [42].

There is controversy regarding how TT affects overall prognosis. Kaptein et al. found that the presence of RCC-TT relative to RCC resulted in a 1.65 increased hazard of death [14]. Other studies have attempted to estimate overall survival (OS) for RCC-TT patients. Chen et al. found a three and five year OS of 58% and 39% respectively [49]. An international cohort of 1,122 patients with RCC-TT noted a median OS of 33.8 months [6] Klatt et al. noted a disease-specific survival for RCC-TT of 36% at five years and 24% at 10 years [50]. Geographic region has also been shown to impact survival, and in our previous publication we analyzed OS and cancer-specific survival (CSS), noting South Korean patients had a greater OS and CSS relative to patients receiving their care in CSA and NA [17]. The literature also varies regarding how TT level changes survival. Klatte et al.’s study noted that a greater TT level increased perioperative morbidity and mortality associated with RN-TT but not OS [50]. The original Libertino classification system found no difference in survival by TT level in the IVC but that in comparison to renal vein TT, patients with an IVC TT had a lower 10 year OS [51]. A similar Japanese study spanning 1995–2013 found extension of TT into the IVC to be associated with worse OS [16]. Wagner et al.’s study comprising 13 different institutions and nearly 1,200 patients found that there was a difference in OS between renal vein involvement versus IVC involvement, however there was not a difference related to TT level within the IVC [18]. These studies contrast with Martinez-Salamanca et al.’s study where a greater TT level was associated with worse OS, and each increase in TT level lowered the OS [6]. There is not strong evidence to predict the development of mRCC-TT or recurrence. However, Pieretti et al. performed a study on 224 patients who underwent RN-TT, and approximately 39% of patients had recurrence postoperatively [52]. In our own publication, we found 47% of patients from a large international cohort became metastatic after RN-TT [17].

## Metastasis

The overall rate of mRCC at diagnosis ranges, but approximately one third will be metastatic at diagnosis [53]. Further, 20–50% of patients may eventually become metastatic despite surgical treatment [53]. Metastasis have also been shown to vary by tumor size, and the rate of metastasis goes up as tumor size increases [54]. Aside from tumor size, presence of TT have independently been proven to be associated with a greater likelihood of developing metastases [14]. There is not strong evidence on the prevalence of mRCC-TT. Somewhere between 28–56% of mRCC patients will have vascular invasion with a TT though [17, 38, 55–57].

Cytoreductive nephrectomy (CN) for mRCC is a controversial topic. The CARMENA and SURTIME trials examined CN followed by ST versus ST alone with tyrosine kinase inhibitor (TKI) [58, 59]. These studies showed that upfront CN did not improve OS and questioned a long-held dogma in urology that upfront CN is beneficial for mRCC patients. There is very little prospective data for CN for mRCC-TT (CN-TT). Mittal et al. performed a retrospective analysis of mRCC patients who either underwent CN-TT or ST only [55]. Forty-six patients with mRCC-TT underwent CN-TT plus ST and 18 underwent ST alone. Median OS was better in patients who underwent CN-TT compared to ST (29.4 months versus 12 months). Interestingly, TT level did not impact OS in this analysis [55]. Goetzl et al. published a 33 patient retrospective analysis on CN, of which 33 underwent CN-TT [56]. In this series, 73% of CN-TT cases were symptomatic at diagnosis. Regarding TT level, 64% had a TT limited to the renal vein, 30% limited to the IVC below the diaphragm, and 6% with a TT extending into the IVC and above diaphragm [56]. Approximately 40% of patients had metastasis limited to the lung. The two and five-year OS was 24.1% and 4% respectively, with no difference by TT level [56]. Of note, the intraoperative complication rate was 14.2%, which is similar to other publications noting an intraoperative complication rate of 16% [56, 60]. Lenis et al. performed a National Cancer Database study of mRCC patients, of which 2,334 were identified as having mRCC-TT [57].

In our own data with ICORCC, we have accumulated a large series of CN-TT [61]. This data is not fully analyzed yet, however there are interesting results worth discussing. Our current series stems from ICORCC and includes 131 patients who underwent CN-TT spanning from 1999–2024 [61]. Using the Neves and Zincke classification system in our series, 56 (44%) patients had a level I TT, 31 (24%) had a level II TT, 20 (16%) had a level III TT, and 20 (16%) had a level IV TT. Fifty percent of patients had metastasis at the lungs, 12% had metastasis in bone, 13% had liver metastasis, 2% had brain metastasis, 24% had retroperitoneal lymph node metastasis, 7% had paraaortic lymph node metastasis, 10% had adrenal metastasis, and 10% had metastasis at “other sites.” Median OS was 1 year, and median CSS was 1 year after CN-TT. Fifty-nine (55%) patients who underwent CN-TT experienced either disease progression or recurrence postoperatively at a median time of four months. Regression analysis was also performed to identify risks for death after CN-TT, and liver metastasis predicted a greater hazard of death based on preliminary analysis [61]. We feel that a critical question still unanswered is to define which patients benefit from CN-TT. Interestingly, prior evidence has shown the liver metastasis is associated with worse OS in RCC, which our preliminary findings seem to align with, while recognizing additional formal analysis is required to make more definitive conclusions [62, 63].

An additional question worth considering is whether surgical resection in the form of RN-TT should be considered the gold standard for mRCC-TT patients. As noted, there is currently limited evidence to predict OS postoperatively from CN-TT. Abel et al. published a series of 427 patients with mRCC-TT that underwent CN-TT [64]. The authors found that poor OS postoperatively was associated with extension of TT above the diaphragm, presence of systemic symptoms, sarcomatoid differentiation, and poor metastatic risk grouping. They concluded by noting that ST may have a role in the patients who have poor prognostic indicators for OS after CN-TT [64]. Miyake also reported a case series of 75 mRCC-TT patients who underwent CN-TT [65]. Liver metastases predicted worse OS. They also analyzed ST administration after CN-TT, finding administration of cytokine therapy alone was associated with worse OS. The future of surgical management for mRCC-TT then, is likely intertwined with ST.

## Systemic Therapy

The benefits of ST for RCC are well-reported. Multiple landmark randomized clinical trials (RCTs) have been published that have provided a paradigm shift in the way RCC is managed, most specifically mRCC [66–70].

ST in RCC-TT patients is more difficult to study because of a significantly smaller population size than mRCC. As noted in other publications, the lack of prospective data and focus on retrospective data on ST in RCC-TT limits the clinical value and practice patterns often do not shift [71]. There are, however, two relevant ongoing clinical trials on ST for RCC-TT worth discussing and one prior published study. The best prospective study currently published is the NEOTAX study, which assessed neoadjuvant (NAT) toripalimab plus axitinib in clear cell RCC-TT with IVC TT [72]. The trial enrolled 25 patients, of which 11 (44%) had a reduction in TT level using the Mayo Clinic classification system. Additionally, no patients saw an increase in their TT level [72]. One ongoing trial is the NAT pembrolizumab and axitinib in renal cell carcinoma with associated inferior vena cava tumor thrombus (NEOPAX) trial (NCT05969496) [73]. This is an open-label phase two clinic trial enrolling patients with clear cell RCC-TT. Patients will receive 12 weeks of axitinib and pembrolizumab with RCC-TT afterwards. The primary study outcome is a change in IVC TT extent using the Mayo Clinic classification system, using MRI imaging obtained at baseline and at the completion of ST (12 weeks) [12]. An additional primary outcome is the change in IVC TT size from baseline imaging [73]. The second trial is the safety and efficacy of NAT lenvatinib and pembrolizumab in patients with renal cell carcinoma and IVC tumor thrombus (NCT05319015) [74]. This is an open-label phase 2 study currently enrolling patients with histologically confirmed cT3-4, N0-1, M0-1 RCC of any subtype with a level II-IV IVC TT. Enrolled participants will receive NAT levantinib (20 mg daily) and pembrolizumab (200 mg every three weeks) for 12 total weeks prior to RCC-TT for locally advanced disease. There are multiple primary study endpoints, which are disease control rate, local and metastatic progression, as well as 90-day postoperative complications [74].

The majority of ST information on RCC-TT comes from retrospective studies. Most research has examined ST’s effect on TT/tumor size. Moinard-Butot performed a 44 patient retrospective study of RCC-TT patients receiving NAT IO for their disease, and of these 86% were mRCC-TT [75]. ST was used preoperatively. There were 11 (25%) patients who achieved a partial response and one patient with a complete response. Further, 20 (45%) patients had progressive disease, 12 (28%) had stable disease, and the total disease control rate was 55% [75]. Immune checkpoint inhibitors (ICIs) were the focus of this study, and on sub-analysis, ICIs showed a response rate against TT of 38% [75]. Suzuki et al. reviewed 68 patients with RCC-TT, all of which had right sided tumors and a TT ≥ level II [76]. The authors compared 23 patients that had presurgical ST to 45 without ST. In the ST cohort, 48% of patients had a reduction in TT size. Further, ST reduced operative times, intraoperative blood loss, the need for extracorporeal circulation, the incidence of ≥ Clavien-Dindo III complications, and length of stay after RN-TT [76]. Hara et al. studied 31 RCC-TT patients without NAT ST and compared them to 19 patients that received NAT ST [77]. The NAT ST grouping showed a decrease in primary renal tumor size and neutrophil-to-lymphocyte ratios compared to the group without NAT ST. Conversely, Bigot et al. reviewed 14 patients that underwent NAT therapy for clear cell RCC-TT, and found limited to no beneficial effect of ST on tumor size reduction [78]. Gu et al. performed a systematic analysis of studies examining the role of NAT ST for RCC-TT [72]. There were 204 patients that underwent NAT ST in the analysis, which included 29 single-arm studies and 5 cohort studies. The total reduction rate in TT level was 29.4%, and the final conclusion was that NAT ST is safe and feasible in RCC-TT [72].

While most work on ST for RCC-TT has focused on a reduction of TT in either size or level, additional questions about patient survival exist. In our own analysis from ICORCC, we found that OS and CSS improved in patients that received preoperative ST prior to CN-TT relative to mRCC-TT patients that did not receive ST [79]. In Hara et al.’s analysis, OS and disease-free survival significantly improved in the group receiving NAT ST [77]. Zisman et al. studied 207 RCC-TT patients as well as 607 RCC patients, and focused on the role of CN and IO [15]. In total, 130 patients had metastatic disease, and when CN was performed, TT did not appear to influence response to IO. Specifically focusing on RCC-TT, the authors also found the mean 2-year OS to be greater in patients who received IO then RN-TT compared to RN-TT alone as well as IO alone [15].

There is even less research on postoperative ST for RCC-TT, which can be administered in a planned adjuvant setting after RN-TT, or for recurrences/metastases postoperatively. Gu et al. performed a prospective cohort trial on adjuvant sorafenib and sunitinib for non-mRCC-TT patients [80]. There were 147 total patients, of which 27 received sorafenib, 17 sunitinib, and the rest were controls. Disease-free survival nor OS significantly differed between groups [80]. We have also analyzed the role of postoperative ST after RN-TT from ICORCC data. This analysis failed to identify any OS, CSS, or MFS benefit [79]. Another potential role of ST is in the salvage setting for recurrences or metastases after RN-TT. Faria-Costa et al. published a 15-year experience of 64 patients with RCC-TT patients who underwent RN-TT [81]. No patients in this series received true adjuvant ST, and rather only underwent ST for recurrences or metastasis. There were 27 (42%) patients with a recurrence and of these 18 (67%) received ST for recurrences and 6 (22%) for metastasis. The median PFS was 23 months, and one-year and five-year PFS were 64% and 32%, respectively [81].

**Fig. 2:**
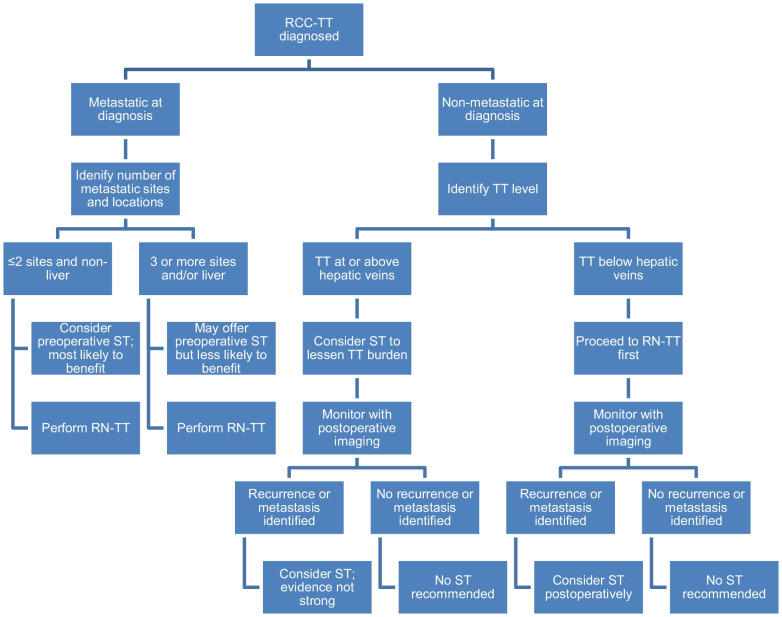
Systemic therapy algorithm. The following is an algorithm for providers to utilize when considering the use of ST for RCC-TT patients. The justification for this algorithm is based off anecdotal evidence of the authors of this manuscript and is meant to guide decision-making but is not level I evidence

In our experience treating RCC-TT patients and based on review of the literature, we have developed an algorithm for practitioners to utilize in deciding whether to consider ST for treating the disease. This is shown in Fig. 2.

## Conclusions

While significantly less common than RCC, there is still a sizeable portion of patients who will present with RCC-TT. These patients may present as symptomatic with systemic or obstructive symptoms, but often RCC-TT is incidentally diagnosed. It is critical to adequately classify the level of the TT, and there are a multitude of systems which can be used to do this. No specific classification system has been definitively proven to be superior. RN-TT is the gold standard treatment, but there is not a comprehensive understanding yet of why TT forms in certain patients nor why the genomic profile of a TT often differs from the primary RCC tumor of origin. mRCC-TT patients often have poor prognosis, and the role of CN-TT needs to be further defined. ST may be beneficial, but the specific patient population in RCC-TT who should receive it also lacks a strong definition. Due to RCC-TT being relatively uncommon, prospective trials are difficult to perform. This further highlights the need for multi-institutional collaborative efforts to study the disease. In this narrative review, we highlight areas requiring further research, and hope this information leads to better patient outcomes in RCC-TT for all.

## Key References

- Neves RJ, Zincke H. Surgical treatment of renal cancer with vena cava extension. *Br J Urol* 1987; 59: 390–395.
  - Original classification system of renal cell carcinoma tumor thrombus.
- Moinzadeh A, Libertino JA. Prognostic significance of tumor thrombus level in patients with renal cell carcinoma and venous tumor thrombus extension. Is all T3b the same? *J Urol* 2004; 171: 598–601.
  - Important classification system of renal cell carcinoma tumor thrombus.
- Mandhani A, Patidar N, Aga P, et al. A new classification of inferior vena cava thrombus in renal cell carcinoma could define the need for cardiopulmonary or venovenous bypass. *Indian J Urol* 2015; 31: 327–332.
  - Important classification system of renal cell carcinoma tumor thrombus.
- Gu L, Peng C, Liang Q, et al. Neoadjuvant toripalimab plus axitinib for clear cell renal cell carcinoma with inferior vena cava tumor thrombus: NEOTAX, a phase 2 study. *Signal Transduct Target Ther* 2024; 9: 264.
  - Clinical trial published on systemic therapy for renal cell carcinoma with a tumor thrombus.
- Sandberg M, Vancavage R, Marie-Costa C, et al. A comparison of renal cell carcinoma with tumor thrombus across North America, Central/South America, and South Korea. *Arch Ital Urol Androl* 2025; 13,820.
  - Geographical information on renal cell carcinoma with a tumor thrombus.
- Sharma S, Kunc M, Czapliński M, et al. Biology of renal cancer tumor thrombus—towards the personalized approach. *Crit Rev Oncol Hematol* 2025; 211: 104,731.
  - Important paper on pathophysiology of tumor thrombus.
- Abel EJ, Spiess PE, Margulis V, et al. Cytoreductive Nephrectomy for Renal Cell Carcinoma with Venous Tumor Thrombus. *J Urol* 2017; 198: 281–288.
  - Important paper on cytoreductive nephrectomy for renal cell carcinoma with a tumor thrombus.

## Data Availability

Data regarding this manuscript is not publicly available secondary to patient privacy but is available in de-identified format upon reasonable request from the corresponding author.
